# Chemical and biological work-related risks across occupations in Europe: a review

**DOI:** 10.1186/1745-6673-9-28

**Published:** 2014-07-24

**Authors:** Diego Montano

**Affiliations:** 1 Faculty of Medicine, Senior professorship “Work Stress Research”, Duesseldorf University, Universitaetsstr. 1, Duesseldorf, Germany

**Keywords:** Work-related diseases, Occupational diseases, Chemical and biological occupational hazards, Social determinants of health

## Abstract

**Background:**

Work-related health inequalities are determined to some extent by an unequal exposure to chemical and biological risk factors of disease. Although their potential economic burden in the European Union (EU-25) might be substantial, comprehensive reviews focusing on the distribution of these risks across occupational groups are limited. Thus, the main objective of this review is to provide a synopsis of the exposure to chemical and biological hazards across occupational groups. In addition, main industrial applications of hazardous substances are identified and some epidemiological evidence is discussed regarding societal costs and incidence rates of work-related diseases.

**Methods:**

Available lists of carcinogens, sensitisers, mutagens, reprotoxic substances and biological hazards were consulted. For each work-related hazard the main industrial application was identified in order to assess which ISCO occupational groups may be associated with direct exposure. Where available, information on annual tonnage production, risk assessment of the substances and pathogens, and other relevant data were collected and reported.

**Results:**

Altogether 308 chemical and biological hazards were identified which may account to at least 693 direct exposures. These hazards concentrate on the following major occupational groups: technicians (ISCO 3), operators (ISCO 8), agricultural workers (ISCO 6) and workers in elementary occupations (ISCO 9). Common industrial applications associated with increased exposure rates relate among others to: (1) production or application of pigments, resins, cutting fluids, adhesives, pesticides and cleaning products, (2) production of rubber, plastics, textiles, pharmaceuticals and cosmetics, and (3) in agriculture, metallurgy and food processing industry, Societal costs of the unequal distribution of chemical and biological hazards across occupations depend on the corresponding work-related diseases and may range from 2900 EUR to 126000 EUR per case/year.

**Conclusions:**

Risk of exposure to chemical and biological risks and work-related disease incidence are highly concentrated on four occupational groups. The unequal burden of exposure across occupations is an important contributing factor leading to health inequalities in society. The bulk of societal costs, however, are actually being borne by the workers themselves. There is an urgent need of taking into account the health impact of production processes and services on workers’ health.

## Background

Physical, chemical and biological risks still account for a substantial proportion of work-related diseases and fatalities in Europe [1]. Frequently, these risks are associated with chronic illnesses such as cancers, allergies and musculoskeletal disorders whose pathogenesis involve in most cases long exposure periods. However, official occupational disease data comprises generally those illnesses only for which there is an unequivocal causal chain from occupational exposure to disease occurrence (e.g. mesothelioma caused by asbestos exposure). For the majority of work-related diseases whose causal pathways are multifactorial, adequate data are actually scarce [1,2].

What is known is that despite great advances in occupational health surveillance in European countries in the last decades, adverse health outcomes are still more frequently observed among workers in hazardous occupations [3]. Moreover, previous epidemiological evidence has shown that work-related health inequalities are determined to a large extent by an unequal exposure to risk factors of disease [4,5]. The potential economic burden of work-related health inequalities in the European Union (EU-25) might be substantial. For the year 2004 only, the estimated productivity and income costs associated with health inequalities amounted to 141 billion EUR (1.35% of EU-25 GDP), whereas the estimated morbidity and mortality costs were 208 billion EUR (2.68% of EU-25 GDP) and 700 billion EUR (6.7% of EU-25 GDP), respectively [6].

Furthermore it is clear that a proportion of diseases and corresponding economic costs is due to the unequal burden of exposure to chemical and biological hazards across occupations. For this reason, it is necessary not only to update and expand our knowledge on the etiological mechanisms associated with those hazards but also to identify the distribution of exposure across occupations in order to reduce more effectively the resulting work-related health inequalities [7]. Although there is substantial research on specific chemical and biological hazards, comprehensive reviews focusing on the distribution of these risks across occupational groups are limited and challenging at the same time due to the different properties of compounds and pathogens, and the complex pathogenesis of the diseases involved (see e.g. [8,9]).

In this context, the purpose of this review is to provide a synopsis of the distribution of specific chemical and biological risks across occupations. Besides other factors such as education, living conditions and income, these risks may contribute substantially to the causal mechanisms leading to work-related health inequalities. In order to fulfil the main objective the following tasks were undertaken: (1) identification of the occupational groups that may be at higher risk of exposure to specific carcinogens, sensitisers, mutagenic, reprotoxic and biological hazards, (2) identification of common occupational settings and industrial applications of hazardous substances, and (3) synthesis of some epidemiological evidence regarding the societal costs, assessment, incidence or methodological problems associated with those hazards.

## Methods

### Chemical hazards

#### Carcinogens

The identification of carcinogenic agents was based on the IARC classification and corresponding IARC-Monographs [10-12]. Only agents belonging to group 1 were considered, i.e. agents for which there is sufficient evidence of carcinogenicity in humans, and therefore, a causal relationship between agent and increased incidence of malignant neoplasms has been established. The estimates of the number of specific exposures of a worker were taken from the database CAREX (Carcinogens Exposure) [13]. These estimates correspond to the exposure period 1990–1993 across 55 industrial sectors for the EU-15 countries. Notice that exposures in the database CAREX refer neither to the number of exposure events nor to the number of workers exposed, but to the occurrence of specific exposures of an individual worker. Additionally, the estimated cancer-related deaths in the United Kingdom for the year 2005 were considered [14].

#### Sensitising, mutagenic and reprotoxic substances

The identification of sensitising substances was based on the list of compounds published in 2013 by the German Commission for the Investigation of Health Hazards of Chemical Compounds (MAK-Commission) [15,16]. Sensitising substances, i.e. substances capable of inducing an immunological response to an otherwise innocuous antigen [17], are classified either as “Sa”, “Sh”, or “SP”. The label “Sh” designates substances that can cause allergic or irritant reactions of the skin and the mucosa close to the skin (skin-sensitising) such as irritant contact dermatitis (ICD), allergic contact dermatitis (ACD), protein contact dermatitis (PCD), and contact urticaria (CU) [18,19]. The label “Sa” designates substances causing airway sensitisation. These involve allergic reactions such as bronchial asthma or rhinoconjunctivitis, and other effects associated with systemic reactions (anaphylaxis). The label “SP” designates substances causing photocontact sensitisation, i.e. an allergic reaction of the skin due to the interaction of the substance with ultraviolet radiation [20]. In general, the classification of a substance as sensitising is based on either sufficient empirical evidence of allergenic and/or irritant effects, or in cases where the allergenic effect can be considered probable on the basis of appropriate empirical evidence.

Most of the mutagenic (M) and reprotoxic substances (R) (i.e. toxic to reproduction) were taken from the German Technical Rules for Hazardous Substances 905 (TRGS 905) updated May 2008 [21]. In order to complement the information on reprotoxicity those substances associated with developmental toxicity reported in the list of the German MAK-Commission were included [15]. According to Annex 6 of the Council Directive 67/548/ECC mutagenic substances refer to substances giving rise to an enhanced occurrence of genetic mutations that may be transmitted to the offspring, i.e. permanent changes in the amount of the genetic material resulting in a change of the phenotypic characteristics of the organism and its offspring. Substances toxic to reproduction refer to substances causing either impaired fertility (“RF”) or subsequent developmental effects in the progeny (“RE”). Mutagenic and reprotoxic substances are classified in categories 1, 2 or 3 according to Annex 6 of the Council Directive 67/548/ECC. In general, for the substances in category 1 there is sufficient evidence of a causal association between human exposure to those substances and heritable genetic damage or impaired fertility, respectively. Substances in category 2 should be regarded as if these were mutagenic or reprotoxic. For substances in category 3 there is some probability of mutagenic or reprotoxic effects. In Section B of the Additional file 1, however, only those substances classified either in category 1 or 2 are considered. Substances associated with developmental toxicity (RE) obtained from the list of the MAK-Commission are evaluated by a different toxicity level scheme. The MAK-Commision recognises four pregnancy risk groups: (i) *Group A*: damage to the embryo or foetus in humans has been unequivocally demonstrated and is to be expected even when occupational exposure limits are observed; (ii) *Group B*: damage to the embryo or foetus cannot be excluded after exposure to concentrations at occupational exposure limits, (iii) *Group C*: there is no reason to fear damage to the embryo or foetus when occupational exposure limits are observed, and (iv) *Group D*: either there are no data for assessment of damage or the currently available data are insufficient for classification in one of the groups A to C. In this review reprotoxic substances belonging to group B only are included.

### Biological hazards

The identification of biological hazards across ISCO categories was based on the systematic review published by J. Haagsma and collaborators [22]. The assessment of infection risk of these hazards was taken from the list of pathogens included in the Directive 2000/54/EC on the protection of workers from risks related to exposures to biological agents at work. According to this directive, biological agents include only cellular or non-cellular microbiological entities capable of replication and of provoking infection or other diseases. Concerning the risk of infection, biological agents are classified into four risk groups depending on the strength of causal relationship between exposition and disease. In general, group 1 includes agents that are not likely to cause human disease; group 2 means that the agent can cause disease; in group 3 agents can cause severe disease and are a serious hazard to workers. Agents in group 4 can also cause severe disease and are a serious hazard to workers, but there is usually no effective prophylaxis or treatment available for them. Even though this classification pertains to the risk of infection only, the directive explicitly states the need of including allergenic and toxigenic effects in the risk assessment of biological agents. It should be remarked here that organic materials, plants, organic dusts, animals (excluding endoparasites) or other vegetal and animal products are not considered biological agents [23]. Instead, toxic and/or sensitising effects of biological agents relate to the components of the agents, their metabolites or the agents themselves such as endotoxins (lypopolysaccharides present in the outer cell membrane of Gram-negative bacteria which are released once the bacteria cell is dissolved), Gram-positive bacteria such as *Actinomyces* spp., mycotoxins (low-molecular toxic metabolites of molds such as gliotoxin), and high-molecular toxic constituents of the cell membrane of fungi released once the fungus is dissolved (*β*-1,3-glucans) [23,24].

### Information on chemical substances and compounds

All information regarding use and manufacture of chemical substances and compounds related to carcinogenic, mutagenic, reprotoxic and sensitising agents was based primarily on Ullmann’s Encyclopedia of Industrial Chemistry, the Toxicology Data Network, PubChem Compound and Haz-Map databases developed by the US National Library of Medicine, the GESTIS-database on hazardous substances provided by the German Social Accident Insurance (IFA), and the eChemPortal provided by various international organisations [25-29]. Information on the total tonnage band (TTB) of hazardous substances manufactured and/or imported per year to the European Economic Area (EU-27 + Iceland, Norway, Liechtenstein) was obtained by matching the CAS numbers of each substance with the CAS numbers of the substances listed in the REACH database (Registration, Evaluations, Authorization and Restriction of Chemicals - REACH) [30].

### Assignment of ISCO-2008 codes

Since a detailed cross-tabulation of occupational titles and exposure to each carcinogen, sensitiser, mutagen and reprotoxic agent is lacking, major ISCO groups were assigned to each substance. Only the first digit of the ISCO classification was used which forms the basis of the ISCO versions 1988 and 2008. This assignment was performed by taking into account, on the one hand, the major production processes and occupational settings implied in the use and/or manufacture of the agent, and on the other hand, those occupations for which a direct exposure can be assumed. The construction of a detailed job-exposure matrix was not pursued. Instead, the assignment serves only descriptive purposes. The major and sub-major occupational ISCO groups are listed in Table 1. Table 2 summarises for each major ISCO group the number and class of chemical substances to which workers might be exposed.

**Table 1:** ISCO 2008 major and sub-major occupational groups

**ISCO**	**Major group**	**Subgroups**
1	Managers	Chief executives, senior officials and legislators, administrative and commercial managers, production and specialised services managers, hospitality, retail and other services managers,
2	Professionals	Science and engineering professionals, health professionals, teaching professionals, business and administration professionals, information and communications technology professionals, legal, social and cultural professionals
3	Technicians and associate professionals	Science and engineering associate professionals, health associate professionals, business and administration associate professionals, legal, social, cultural and related associate professionals, information and communications technicians
4	Clerical support workers	General and keyboard clerks customer services clerks, numerical and material recording clerks, other clerical support workers
5	Service and sales workers	Personal service workers, sales workers, personal care workers, protective services workers
6	Skilled agricultural, forestry and fishery workers	Market-oriented skilled agricultural workers, market-oriented skilled forestry, fishing and hunting workers, subsistence farmers, fishers, hunters and gatherers
7	Craft and related trades workers	Building and related trades workers, excluding electricians, metal, machinery and related trades workers, handicraft and printing workers, electrical and electronic trades workers, food processing, wood working, garment and other craft and related trades workers
8	Plant and machine operators, and assemblers	Stationary plant and machine operators, assemblers, drivers and mobile plant operators
9	Elementary occupations	Cleaners and helpers, agricultural, forestry and fishery labourers, labourers in mining, construction, manufacturing and transport, food preparation assistants, street and related sales and service workers, refuse workers and other elementary workers
0	Armed forces occupations	Commissioned armed forces officers, non-commissioned armed forces officers, armed forces occupations, other ranks

**Table 2:** Number of chemical and biological hazards to which workers may be exposed in each major ISCO occupational group

**Hazard type**	**ISCO 0**	**ISCO 2**	**ISCO 3**	**ISCO 4**	**ISCO 5**	**ISCO 6**	**ISCO 7**	**ISCO 8**	**ISCO 9**	**Industrial uses or work settings**	**Some implied costs**
Carcinogens	NA	7	23	NA	4	5	6	37	8	Manufacture of rubber, plastics, dyes, steel, inks, textiles, paper and batteries, semiconductors, glass, and cement; in construction activities such as building demolition, roofing, painting, and stonework; in such processes or work tasks involving incomplete combustion of organic material, and mining activities involving nickel, lignite and haematite mining	Annual societal costs of occupational cancer: 126000 EUR per case [34]
Skin sensitisers	7	16	106	1	21	21	10	106	11	In the rubber industry as antioxidants, accelerators, and vulcanization agents, in the production of dyes, epoxy resins, textiles, paints, cosmetics, foods; in the pharmaceutical industry, and the production of pesticides	Annual societal costs of hand eczema: 8798 EUR per case [43]
Airway sensitisers	NA	2	14	NA	NA	8	2	15	1	Production of plastics, paper, resins, textiles, cosmetics, dyes, and in the metallurgic, food and agricultural industry	Annual societal costs of occupational asthma: 2900 EUR per case [69]
Mutagenic (category 2)	NA	NA	5	NA	NA	1	NA	5	1	Production of plastics, textile fibres; as additives and intermediates	Societal costs associated with heritable diseases
Toxic for fertility	1	1	10	NA	1	2	3	11	3	Production of plastics, inks, textiles, pigments, adhesives; as intermediates and solvents	Societal costs of reduced fertility
Toxic for development	1	1	31	NA	3	9	10	32	17	Production of adhesives, textiles, dyes, insecticides, lubricants, varnishes, cutting fluids, cements, cellulose; in the rubber and plastics industry; as solvents	Societal costs of congenital malformations
Biological	1	23	21	NA	18	20	10	4	17	Healthcare, biotechnology, agriculture, forestry, outdoor tasks, military	Societal costs of several infectious diseases
Total hazards	10	50	210	1	47	66	41	210	58	693 exposures to chemical and biological hazards	

NA: Not available

The procedure for assigning the ISCO categories to each hazard can be illustrated with two examples. The carcinogen benzene (71-43-2) is used in the production of plastics, resins, nylon fibres, dyes, detergents, and gasoline. According to the CAREX database most of the workers exposed to benzene are employed in the manufacturing, transport, wholesale and retail trade sectors. The corresponding major ISCO categories would correspond roughly to 3, 5, 8, and 9 respectively. The allergen methyl vinyl ketone (78-94-4) is used as a starting material for plastics and as an intermediate in the synthesis of steroids and vitamin A. Exposure may be expected among workers involved directly with this substance during manufacturing processes. Consequently, workers at risk are expected to be employed in occupations covered by ISCO categories 3 (technicians) and 8 (operators). The assignment of ISCO categories to the pathogens included in Section D of the Additional file 1 was based on the review of J. Haagsma and collaborators [22].

## Results

### Chemical hazards

#### Carcinogens

The list of carcinogenic substances is reproduced in Section A of the Additional file 1. Overall 42 carcinogenic substances belonging to group 1 of the IARC classification were considered. If the estimates of exposures for the period 1990–1993 are taken as reference, at least 35 million exposures to a specific carcinogenic substance can be expected in the EU-15 countries. In the United Kingdom the estimated cancer-related deaths in the year 2005 for some of the substances listed in Section A of the Additional file 1 is approximately 132948. The most frequent cancer sites are lung (19 substances), bladder (12 substances), scrotum (4 substances), paranasal sinuses (3 substances), and skin (3 substances). Commonly, exposure to carcinogenic substances occur in different industrial production processes such as (i) manufacture of rubber, plastics, dyes, steel, inks, textiles, paper and batteries, semiconductors, glass, and cement, (ii) in construction activities such as building demolition, roofing, painting, and stonework, (iii) in such processes or work tasks involving incomplete combustion of organic material such as coal gasification, coke production, and diesel exhaust, and (iv) mining activities involving nickel, lignite and haematite mining.

As a consequence, exposures are concentrated in the ISCO groups associated with tasks usually performed in the craft, manufacturing, construction and mining sectors. From Table 2 it can be seen that technicians (ISCO 3) and operators (ISCO 8) are exposed to at least 23 and 37 substances, respectively. Service workers, agricultural and craft workers, professionals and workers in elementary occupations are exposed to at least 4, 5, 6, 7, and 8 carcinogenic substances, respectively. Moreover, it should be remarked that several elementary occupations usually classified as ISCO category 9 whose work tasks are not directly related to known hazardous production processes may also be at risk (e.g. cleaners, labourers, etc.), if the work environment or industrial setting presupposes increased exposure to carcinogenic substances.

Epidemiological studies suggest that cancer incidence follows the distribution pattern of exposure to carcinogens. The results of a large census-based study on cancer incidence by occupational category in the Nordic countries indicate high standardised incidence rates (SIR) of all malignant neoplasms among men for occupations such as waiters, beverage, tobacco and metal workers, seamen, plumbers, packers, chimney sweeps, sales agents, miners and drivers. Among women high SIRs were observed for occupations such as journalists, clerical workers, construction workers, printers, tobacco workers, transport workers and waiters. In contrast, occupations with a low exposure to industrial agents such as farmers, gardeners and fishermen had the lowest SIRs for all malignant neoplasms (for details see Table eighty in [31]). Rushton et al. [14] estimated the overall burden attributable to selected occupational carcinogens in Great Britain to be 8.2% for men and 2.3% for women, with latency periods ranging from about 10 to 40 years [14]. The attributable fractions (AF) for some cancer sites such as mesothelioma, sinonasal and lung cancers were particularly large ranging from 21% to 97%.

Kogevinas and colleagues [32] confirmed the excess risk of bladder cancer among workers employed in 12 out of 31 occupations suspected of being associated with bladder cancer in a large epidemiological study [32]. These occupations belong almost exclusively to ISCO groups 7 and 8 and include among others knitters, automobile painters and mechanics, metal workers and machinists, textile machinery mechanics, printers, and transport equipment operators. However, given that about 30% of all bladder cancer cases were employed in occupations of the metal sector where aromatic amines are not the predominant exposure (i.e. machinists, transport equipment operators, and miners), PAH, diesel engine exhaust, cutting oils, solvents and metal fumes may account for the excess risk observed [32,33].

Unfortunately, economic analyses of the societal costs of work-related cancer in Europe are limited and difficult to perform given serious methodological difficulties regarding the estimation of attributable fractions and the lack of appropriate data. Nonetheless, Binazzi and colleagues [34] estimated the annual burden of occupational cancer in Italy for the year 2006 to lie between 8000 and 8500 deaths. The corresponding direct costs (i.e. treatment costs) of occupational cancer have been estimated at 456 million EUR or about 57000 EUR per case. Indirect costs resulting from the potential years of working life lost (PYWLL) ranged between 320 and 590 million EUR [34]. In Spain, 9469 work-related cancer deaths were estimated for the year 2004. Indirect costs from PYWLL may lie between 34000 and 62000 EUR per case based on the overall estimates for occupational diseases [35].

#### Sensitising, mutagenic and reprotoxic substances

##### Skin sensitisers (Sh)

The list of sensitising substances is reproduced in Section B of Additional file 1. From a total of 143 sensitising substances at least 100 may cause skin sensitisation. Annual production estimates of substances associated with skin sensitisation (Sh) are quite large. They range between 1 to 10 million (2 substances), 0.1 to 1 million (4 substances), 10 to 100 K (17 substances), and 1 to 10 K tonnes per annum (16 substances). Sh substances are used in a wide variety of industrial applications, particularly in (i) the rubber industry as antioxidants, accelerators, and vulcanization agents, (ii) the production of dyes, epoxy resins, textiles and paints, (iii) the cosmetic, food and pharmaceutical industry, and (iv) the production of herbicides, fungicides and other pesticides. The large number of Sh substances and the wide variety of industrial settings associated with increased exposure to skin sensitisers explain the fact that technicians and operators (ISCO categories 3 and 8) can be potentially exposed to at least 106 different Sh substances. Concerning the remaining ISCO categories it was found that agricultural workers (ISCO 6), service workers (ISCO 5), professionals (ISCO 2), workers in elementary occupations (ISCO 9), craft workers (ISCO 7), armed forces workers (ISCO 0), and clerks (ISCO 4) may be exposed to at least 21, 21, 16, 11, 10, 7 and 1 skin sensitising substances, respectively (see Table 2).

A large body of epidemiological evidence on skin diseases in some European countries indicates that most of all occupational skin diseases (OSD) are due to irritant or allergic contact dermatitis (ICD and ACD, respectively) localised mostly on the hands and face [36]. ICD is commonly associated with frequent use of water, soaps and detergents, alkalis, acids, metalworking fluids, organic solvents, petroleum products, oxidising or reducing agents, animal products or physical factors such as friction [37]. On the other hand, the most common substances associated with occupational ACD are biocides, chromate, dyes, epoxy resins, fragrances, formaldehyde, (meth)acrylates, nickel, plants and woods, and rubber-processing chemicals [37]. The overall incidence rates of OSD across occupational groups have been estimated for several European samples. The incidence of OSD in Northern Bavaria (Germany) between 1990 and 1999 have resulted in 6.7 cases per 10000 workers [38]. In France, the incidence estimates from 2004 to 2007 was 1.5 per 10000 salaried workers [39], whereas in Spain, the incidence of registered OSD for the year 2006 was 0.7 per 10000 workers [40]. Since available incidence estimates depend on national legislation concerning reporting schemes of OSD and divergent compensation criteria, it has been acknowledged that incidence of OSD is being actually underestimated [36].

The incidence of OSD across occupational groups is rather consistent and corresponds to the increased exposure risks of specific occupational groups. Hairdressers and barbers show the largest incidence of OSD both in Germany and the UK with 97.4 and 11.6 cases per 10000 workers, respectively, followed by bakers and printers [38,41]. Other occupational groups in those two countries showing large incidence rates are workers in the metallurgical industry (electroplaters, machine tool operatives, metal processors, machine setters and operators), the construction industry (tile setters, terrazzo workers, painters, brick layers, cement workers) the food industry (cooks, catering assistant), and the health care sector (dental nurses, dental technicians, nurses) [38,41]. A similar pattern of incidence of OSD can also be observed in France for the period 2004–2007. The highest incidence rates per 10000 workers were observed in the metallurgical industry (1.22), the construction industry (5.29), the chemical and rubber industry (1.97), and the services activities including hairdressers and household workers (1.87) [39]. Regarding the prevalence of OSD, the EU-OSHA 2008 report indicates that the proportion of OSD is highest among craft workers (ISCO 7, 33%), followed by workers in elementary occupations (ISCO 9, 22%), operators (ISCO 8, 14%), and service workers (ISCO 5, 18%) [42].

Although research on societal costs of occupational skin diseases is rather limited, the direct and indirect costs of occupational hand eczema in Germany have been estimated in a study conducted in 2013 by Diepgen and colleagues [43]. The annual direct and indirect costs of occupational hand eczema per worker diagnosed and treated were on average 2646 EUR (95% CI 2265–3027 EUR) and 6152 EUR (95% CI 4508–7797 EUR), respectively. In Italy, the societal costs of severe chronic hand eczema refractory to standard therapy amounted in average to 5016 EUR per person-year (min. 411 EUR, max. 27648 EUR) [44]. Additional costs may also occur in cases of occupational retraining, job change, or adverse psychosocial effects [36]. Moreover, since for severe OSD more than a half of all cases may become persistent [37], that is, they develop a persistent dermatitis even after removal from exposure to causative agents [45], both direct and indirect costs are expected to increase depending on the degree of disability.

##### Substances causing airway sensitisation (Sa)

From the 143 sensitisers in Section B of the Additional file 1 a total of 32 are associated with respiratory sensitisation. Interestingly, from these 32 sensitisers 17 are both skin and airway sensitisers (Sah). The available annual production estimates of substances causing respiratory sensitisation are not as large as the estimates of the skin sensitising substances. Annual production of Sa substances range from 0.1 to 1 million (1 substance), 10 to 100 K (3 substances), and 1 to 10 K tonnes per annum (4 substances). Sa substances are widely used in several industrial processes such as the production of plastics, paper, resins, textiles, cosmetics, dyes, and in the metallurgic and food industry. Sa substances include also biological agents such as animal hair or other materials derived from animals, and cereal flour dusts. Occupations at higher risk of developing allergic reactions to Sa substances are technicians and operators who might be exposed to at least 14 and 15 different Sa substances, respectively. Agricultural workers, craft workers, professionals and workers in elementary occupations may have increased exposure for at least 8, 2, 2, and 1 Sa substances (see Table 2).

Respiratory sensitisers are associated with several respiratory diseases including irritation of mucous membranes, asthma, chronic bronchitis, chronic obstructive pulmonary disease and rhinitis [46,47]. Asthma is one of the most frequent occupational diseases in Europe [48]. Several epidemiological studies have estimated that occupational agents may account for about 5% - 25% of new asthma cases among workers of different industries [49,50]. In spite of the large health and socio-economic impact of occupational asthma, incidence and prevalence are still underestimated [50-52]. Moreover, some studies have pointed out that the number of workers with pre-existing asthma may be even larger and experience a worsening of symptoms [49]. For several European countries the incidence of occupational asthma per 10000 workers has resulted in 0.23 in Belgium (2000–02) [53], 0.24 in France (1996–99) [54], 0.32 in the United Kingdom (1992–97) [55], 1.74 in Finland (1989–95) [56] and 0.28 in Germany (2003) [46].

Incidence rates estimates of occupational asthma from several European countries report an unequal distribution of cases across occupational groups. Assuming that genetic susceptibility is randomly distributed across occupational groups, the unequal distribution of occupational asthma cases is related primarily to (i) the presence or absence of a latency period of airway obstruction associated mostly with allergic (IgE mediated) or irritant-induced asthma, respectively, (ii) the agent to which workers are exposed (low- vs. high-molecular-weight > 5 kDa), (iii) the duration of exposure and the agent concentration [57,58].

In France, the United Kingdom, Sweden and Finland bakers and painters show the largest incidence rates per 10000 workers ranging from 6.8 in France to 44.8 for male bakers in Finland, and from 3.3 in France to 22.3 for male painters and lacquerers in Finland [54-56,59]. The excess risk for bakers can be explained by continuous airway exposure to high-molecular weight agents such as vegetal proteins in cereals, flours, or other protein additives, and enzymes used for controlling the production process (e.g. *α*-amylase) [58]. The excess risk for painters has been associated with low-molecular-weight diisocyanates used in the production of lacquers and spray paints (e.g. 1,6-hexamethylene diisocyanate (822-06-0) and 1,5-naphthylene diisocyanate (3173-72-6)).

In general, workers in the chemical, plastic and metallurgic industries are exposed primarily to low-molecular-weight agents such as metal compounds (e.g. platinum and nickel compounds, tungsten carbide, cobalt) and reactive chemicals (e.g. diisocyanates and anhydrides) which have extensive applications as resins, dyes, production of polyurethanes, adhesives, insulating foams, lacquers and metal alloys for welding. The risk for healthcare workers and cleaners is related to increased exposure to biocides (e.g. glutaraldehyde) and cleaning products (e.g. quaternary ammonium compounds [60]), antiobiotics (e.g. penicilins, psyllium), and natural rubber latex [61]. Dusts of different species of wood associated with occupational asthma (particularly Western red cedar *Thuja plicata*) are the main exposure for sawmill workers, carpenters and related wood processing workers showing relatively large incidence rates [62]. The fact that incidence estimates and occupations at high risk are rather similar for males and females in Finland suggests that the specific exposure mechanisms and the job tasks defined by each occupation should account for most of the gender differences observed [56].

A further complication of exposure to airway sensitisers is the fact that the majority of workers diagnosed with occupational asthma also suffer from occupational rhinitis, an inflammatory disease of the nose characterised by nasal congestion, variable airflow limitation and/or hypersecretion after exposure to sensitisers at the workplace [63]. Epidemiological data from France suggest that this relationship between occupational rhinitis and occupational asthma is especially frequent when high-molecular-weight sensitisers are involved [64]. Despite being a common condition epidemiological data of occupational rhinitis is rather scarce. Some studies have reported larger incidence rates for occupational rhinitis than occupational asthma [63]. Age-standardised rate ratios (SRR) of occupational rhinitis estimated with register data of Finland for the period 1986–91 identified furriers (30.0), bakers (22.0), livestock breeders (22.0), food-processing workers (13.0), veterinarians (11.0), agricultural workers (8.3) and assemblers of electronic products (7.7) as occupations at high risk [65].

Occupational asthma has a poor prognosis; once a worker develops occupational asthma after a latency period the chances of recovery are small [66]. It has been estimated that approximately 70% of workers diagnosed with occupational asthma show symptoms and airway hyperresponsiveness even several years after complete cessation of exposure [67]. Even though complete avoidance of exposure to the allergen is considered the treatment of choice [68], the socio-economic consequences for workers and society are large. Ayres and collegues [69] estimated the direct and indirect costs of occupational asthma in the United Kingdom from data of the Survey of Work-related and Occupational Respiratory Disease (SWORD) [69]. The average direct costs per annum per case range from £530 to £715, whereas the indirect costs range from £1525 to £1685. The total present value costs of an average case to society lies between £120000 and £130000 per annum. Assuming that about one-third of cases is not being diagnosed, the authors estimated that the total lifetime costs of new cases to society from all potential cases in 2003 could lie between £95 and £135 million. Moreover, further analyses revealed that about 49% of the present value total costs are borne by the individual, 48% by the state and only 3% by the employer.

#### Mutagenic and reprotoxic substances

The list of some mutagenic and reprotoxic substances across occupational groups is reproduced in Section C of the Additional file 1. A total of 47 substances are included. Substances classified in category 2 include 5 mutagenic substances (M), 11 substances impairing fertility (RF), and 6 substances with developmental toxicity (RE). Lead and second-hand tobacco smoke only are classified in category 1 of substances causing developmental toxicity (RE), since evidence of an association between exposure to these agents and the risk of adverse pregnancy outcomes such as preterm birth and low birth weight is consistent [70-72]. The most common industrial uses of mutagenic and reprotoxic substances are related to the manufacture and/or use of adhesives, resins, additives, coatings, pigments, inks, polymers, papers, organic solvents, pesticides and woods and textiles.

Regarding occupational exposure to mutagenic substances of category 2, technicians (ISCO 3) and operators (ISCO 8) each are exposed to at least 5 substances, whereas professionals (ISCO 2), agricultural workers (ISCO 6), and workers in elementary occupations (ISCO 9) each to at least 1 substance (see Table 2). For substances impairing fertility in category 2, technicians and operators are exposed to at least 10 and 11 reprotoxic substances respectively, craft workers and workers in elementary occupations each to at least 3 substances, agricultural workers to at least 2 substances, and service workers, professionals and armed forces workers each to at least 1 substance. With respect to substances associated with developmental toxicity (RE) a similar concentration of exposures in ISCO groups 3 and 8 can be observed.

Concerning the risk level B of substances associated with developmental toxicity RE (see Methods section) a total of 26 substances were identified. The most frequent industrial applications are in the manufacture and/or use of adhesives, lacquerers, cements, pesticides, leather, resins, rubber, solvents and plastics. Hence, occupational exposure is expected to occur mostly in technicians and operators (26 substances each), workers in elementary occupations (19 substances), and agricultural and craft workers (8 substances each).

The risk assessment of mutagenic substances poses unique problems in comparison with carcinogens and sensitisers. First, conclusive epidemiological evidence of a causal association between exposure to chemicals or radiations and heritable gene mutations is lacking [15]. This explains the fact that there are no substances classified in category 1 in the table of Section C of the Additional file 1. Second, Singer and Yauk [73] argue that since the mid-1970s research on germline mutation has not been a priority concern compared to the identification of agents that are carcinogenic or mutagenic to somatic cells [73]. The authors identified two major causes limiting the identification of germ cell mutagens in humans so far: (i) the assumption that somatic cell mutagenicity tests also detect germline mutagens (implied also in the REACH Regulation EC 1907/2006, Annexes VIII to X), and (ii) a lack of practical test methods for assessing causal relationships between germline mutations and heritable effects in the offspring.

New developments in mutagenicity research are expected with the introduction of genomics technologies such as high-throughput sequencing analysis [73]. In addition, it has been recognised that epigenetic modifications, i.e. heritable changes in gene expression occurring without changes in DNA sequence, may mediate genotoxicity from chemicals encountered in the environment and occupational settings. Even though it is not yet clear to what extent epigenetic modifications caused by exposure to chemicals induce transgenerational phenotypic effects in humans, some studies have found an association between epigenetic modifications and exposure to nickel, cadmium, methylmercury (22967-92-6), particulate matter in air, benzene, the carcinogen diethylstilbestrol (56-53-1), bisphenol A (80-05-7), persistent organic pollutants and dioxin [74].

Regarding the associations between chemical substances and impaired fertility there are also several challenges for assessing potential hazards. These challenges encompass above all (i) validity issues affecting different endpoints of fertility including time to conception, infertility, and standardised birth ratio, (ii) the ability to control for major confounders of fertility such as drug and alcohol consumption, sexually transmitted infections, etc. (iii) methodological deficiencies of the studies (e.g. suboptimal exposure assessment, recall bias, etc.) [75,76].

However, pooled estimates of the association between occupational exposure to pesticides and fecundability among fruit and greenhouse workers resulted in a substantial reduction of fecundability, especially when considering studies with improved assessment of occupational pesticide exposure [77]. Concerning genotoxic effects of pesticides in germ cells, two meta-analyses confirmed an excess risk of childhodd leukaemia and lymphoma for maternal exposure to pesticides [78,79].

### Biological hazards

The list of biological hazards across occupational groups are reproduced in Section D of the Additional file 1. A total of 50 pathogens are included comprising 25 bacteria (6 belonging to risk group 3), 16 viruses (5 belonging to risk group 3), 7 parasites (1 belonging to risk group 3), and 1 fungus belonging to risk group 3. The distribution of biological hazards across occupational groups differs largely from the distribution of allergens, carcinogens, mutagens and reprotoxic substances discussed in previous sections. In addition, for the majority of biological agents there are still no occupational exposure limits [80]. Professionals (ISCO 2) and technicians (ISCO 3), especially in the healthcare and biotechnology sector (physicians, nurses, dentists, medical residents, microbiologists, medical technicians), are exposed to at least 23 and 20 pathogens respectively, agricultural (ISCO 6) and service workers (ISCO 5), workers in elementary occupations (ISCO 9), craft workers (ISCO 7), operators (ISCO 8), and armed forces workers (ISCO 0) to at least 19, 18, 16, 10, 3, and 1 pathogens, respectively (see Table 2).

Some factors explaining the high concentration of biological hazards in the healthcare and biotechnology sectors and agriculture are related to the nature of work tasks (e.g. patient care, outdoor work, biotechnology), the environmental and the social context in which work is performed (e.g. hospitals, forests, laboratories), and several characteristics of biological agents such as the routes of exposure, the pathogenicity, the mechanisms of transmission (e.g. oral, percutaneous, stings), the mechanisms of dissemination (e.g. water, soil, air), the natural habitat of the biological agents, the particular characteristics of pathogen hosts and/or pathogen vectors and the clinical picture of the disease [81]. From an occupational health perspective, however, airborne and percutaneous transmission play a very important role in risk assessment strategies [24]. First, biological hazards such as bacteria, viruses, fungi, endotoxins and mycotoxins are often present in the form of bioaerosols causing diseases of the respiratory tract, conjunctiva and skin. Second, agents causing infectious diseases can be transmitted by ingestion, vectors such as ticks and mosquitoes or contact skin.

The excess risk of infectious diseases among healthcare workers is related to a large extent to the intense contact with body fluids and tissues, the exposure to airborne agents and percutaneous injuries. Even though great improvements have been achieved in industrialised countries concerning the risk of infection for major diseases such as tuberculosis, hepatitis and influenza in workers and the general population [82,83], healthcare workers are still at a higher risk of infection. The number of new infections of hepatitis B (HBV) and C (HCV) viruses in healthcare workers resulting from sharps injuries alone has been estimated to amount 210 (95% CI 60–730) and 290 (95% CI 100–1600), respectively, for the WHO-European region A in the year 2000 [84]. Estimates of incidence rates of influenza per 100 person/season in working adults in comparison with incidence rates among healthcare workers resulted in 5.44 (95% 3.01–9.84) and 18.69 (95% CI 15.80–22.11) for unvaccinated persons, whereas the incidence rates for the vaccinated were 1.20 (95% CI 0.86–1.68) and 6.49 (95% CI 4.63–9.09) per 100 person/season, respectively [85]. Baussano and collegues [86] found in a comprehensive meta-analysis that the risk of tuberculosis among healthcare workers is consistently higher than the risk among the general population worldwide [86].

In contrast to workers in the healthcare and biotechnology sector, the exposure of veterinarians, farmers and agricultural labourers to biological agents are related to zoonoses, bacterial and parasitic infections and hypersensitivity reactions due to bioaerosols [87]. Farmers and workers in veterinary settings, workers in grain threshing and sieving, flax threshing, herb, composting and wood processing have increased risk of chronic respiratory disorders associated with intense exposure to allergenic microorganisms (e.g. bacteria and moulds) and related pathogen substances, in particular, endotoxins which are associated with non-atopic asthma, bronchial hyperresponsiveness, fatigue, inflammatory reactions, lung function decline and protective effect on allergic sensitisation [24,80,88-91].

Zoonoses, diseases transmitted between animals and humans, account also for a substantial excess risk of disease among veterinarians, agricultural and forestry workers. Among others, tick-borne diseases such as Lyme borreliosis (caused by *Borrelia burgdorferi*), tick-born encephalitis virus and human granulocytic anaplasmosis (caused by *Anaplasma phagocytophilum*), Q fever (caused by infection with *Coxiella burnetii*), and leptospirosis (caused by *Leptospira* spp.) are associated with outdoor activities in agricultural, forestry and veterinarian settings [87,89,92].

The emergence of antimicrobial-resistant bacteria in mass animal husbandry has raised further concern regarding occupational exposure and its possible public health consequences. Particular attention has been paid to methicilin-resistant *Staphylococcus aureus* (MRSA) and mutidrug-resistant Gram-negative enteric pathogens (GNEP). Whereas the prevalence of MRSA in the general population has been estimated to be about 5%, prevalence among equine veterinarians and workers in small-animal hospitals has been estimated to be 10% and 18%, respectively [93].

## Discussion

In this review the distribution of work-related chemical and biological risks across major ISCO occupational groups was summarised. In particular, the following tasks were undertaken: (1) identification of the occupational groups that may be at higher risk of exposure, (2) identification of common occupational settings and industrial applications of hazardous substances, and (3) synthesis of some epidemiological evidence regarding the societal costs, assessment, incidence or methodological problems associated with chemical and biological occupational hazards.

Altogether 308 chemical and biological hazards were identified which may account to at least 693 direct exposures. These hazards, however, concentrate on specific major occupational groups depending on the type of hazard under consideration. The majority of direct exposures are expected among technicians and associate professionals (ISCO 3), operators and assemblers (ISCO 8), agricultural workers (ISCO 6) and workers in elementary occupations (ISCO 9) (see Table 2). Despite the huge variation of industrial processes making use of hazardous chemicals, there are particular applications, occupations or industrial sectors that are commonly mentioned: (1) production or application of pigments, dyes, paints, inks, resins, lubricants, cutting fluids, adhesives, cements, pesticides (fungicides, bactericides, insecticides, viricides) and cleaning products, (2) production of rubber, plastics, textiles (including leather), pharmaceuticals and cosmetics, (3) in agriculture, metallurgy (especially steel and aluminium), and food processing industry, (4) painters, bakers, metal workers, health care workers, hairdressers, wood workers and agricultural workers.

The societal costs of this unequal distribution of chemical and biological hazards across occupations include the direct costs (usually the cost of using healthcare resources) and the indirect costs (usually opportunity costs such as lost income, permanent disability, etc.). Estimates for some important outcomes such as cancer, occupational contact dermatitis and occupational asthma range from 2900 EUR to 126000 EUR per case/year (see Table 2). Since the indirect costs constitute the largest proportion of work-related illnesses, the bulk of societal costs are actually being borne by the workers themselves.

On the basis of these results, the possibilities of prevention and reduction of health inequalities associated with chemical hazards are seriously limited by several facts including (1) the ubiquity of hazardous chemicals in a huge variety of production processes and industrial sectors, (2) the number and production amounts of commonly used chemicals (about 30000 chemicals from 100000 registered [94]), (3) the quantity of hazardous substances registered under the REACH legislation (about 11776 in the year 2014), (4) the costs associated with appropriate toxicological testing (9.5 billion EUR and 54 million vertebrate animals for testing 30000 substances [95]), (5) the costs associated with (bio)monitoring and surveillance systems, (6) the multiplicity of their potential combinations and their effects on health, (7) the uncertainty of etiological mechanisms leading from exposure to disease (especially for mutagens), (8) the wide range of exposure routes, and (9) the deficient compliance of enterprises (particularly of micro-, small and medium-size enterprises) [94].

These challenges point to the urgent need of re-thinking the production process by taking into account the health impact on workers from the very beginning. This is particularly important in case of innovative production processes. The possibilities of eliminating or substituting chemical hazards, and the provision of a health-conducive organisation of work should be given high priority.

### Limitations

This review suffers from several limitations. First, the exposure of managers and clerks has been underestimated (e.g. those working in the production of plastics, rubber, and aluminium). However, the epidemiological evidence summarised in the previous sections suggest that the exposure of managers and clerks is very low in comparison to other occupational groups. On the contrary, since exposure to one or more substances for single occupational titles in the rest of ISCO groups is not an uncommon phenomenon (e.g. for bakers, metal workers, veterinarians, hairdressers, construction workers), it is possible that in fact the inequality of exposures might be even larger. Second, the assignment of ISCO groups is subjected to some extent to misclassification bias since the exact titles of exposed workers are not available for each single chemical and biological hazard. However, the complete lists provided in the Additional file 1 may be easily improved and updated in future studies. Third, the epidemiological evidence discussed was not collected by a systematic query of electronic databases and has therefore a limited scope. Fourth, the identification and selection of chemical and biological hazards was based on available lists from specific institutions (e.g. IARC). Therefore, this review inherits the same methodological deficiencies of the original lists. Finally, only those chemical and biological substances were selected for which there is some evidence of causal relationship between exposure and disease. Thus, overall exposures across and within major occupational groups may have been underestimated. Nonetheless, one important strength of this review lies precisely in its comprehensive approach. The information provided in this paper can be useful for occupational physicians, clinicians and, in general, occupational health specialists working at the company level or interested in expanding the evidence basis of the risks associated with specific chemical and biological hazards. As a general overview, this paper may serve as a first basis to identify some priority areas in future occupational health research.

## Conclusion

Risk of exposure to chemical and biological risks and work-related disease incidence are highly concentrated on four occupational groups (ISCO groups 3, 6, 8 and 9). The unequal burden of exposure across occupations is an important contributing factor leading to health inequalities in society. The bulk of societal costs, however, are actually being borne by the workers themselves. There is an urgent need of re-thinking the production process by taking into account the health impact on workers from the very beginning.

## Competing interests

The author declares that he does not have competing interests.

## Supplementary Material

Additional file 1 **Lists of occupational hazards.** Supplementary tables of carcinogens, sensitisers, mutagens, reprotoxic substances and biological agents by ISCO-2008 major occupational groups. Click here for file
