# Supplementary material for: Chemical and biological work-related risks across occupations in Europe: a review
Source: J Occup Med Toxicol. 2014 Jul 24;9:28. doi: 10.1186/1745-6673-9-28 (PMC4113130; doi:10.1186/1745-6673-9-28)
Supplement: Additional file 1 — Lists of occupational hazards. Supplementary tables of carcinogens, sensitisers, mutagens, reprotoxic substances and biological agents by ISCO-2008 major occupational groups. [file 1745-6673-9-28-S1.pdf]

Chemical and biological work-related risks across occupations in Europe: a review
Supplementary tables

Author:

Diego Montano

Faculty of Medicine

Senior professorship “Work Stress Research”

Duesseldorf University

March 2014

List of Tables

1. Carcinogens of the IARC group 1 . . . 3
2. Sensitising substances . . . 6
3. Mutagenic and reprotoxic substances . . . 15
4. Pathogens by species and risk group . . . 19

A . List of carcinogens

Table 1: Carcinogens of the IARC group 1. Sources: [1, 2, 3, 4, 5, 6, 7, 8]

CAS	Agent	Some uses (or remarks)	ISCO	Cancer site (or type)	Banned in the EU	Exposed EU 1990-3 (thousands)	Deaths UK
	Acid mists, strong inorganic	Production of phosphate fertilizers, isopropanol, synthetic ethanol, lead batteries	2, 8	Larynx	no	710	1494
1402-68-2	Aflatoxins	Toxic metabolite of the common fungus Aspergillus flavus	2, 6	Liver	yes	2	NA
	Aluminium production (exposure to PAH)	Construction, transportation, packaging	8	Bladder, lung	NA	NA	NA
92-67-1	4-Aminobiphenyl	Rubber antioxidant, dye intermediate	8	Bladder	no	NA	558
36465-76-6	Arsenic and inorganic arsenic compounds	Pharmaceuticals, agricultural chemicals, mining, metallurgy	3, 6, 8	Lung, bladder, skin	no	150	2034
	Asbestos (and talc containing asbestiform fibres)	Roofing, themal and electrical insulation, cement pipe and sheets, flooring, friction materials, coating and compounds, plastics, textiles, paper, mastics	3, 6, 8, 9	Lung, mesothelioma, larynx, ovary	yes	1228	70362
	Auramine production	Paper dyeing and flexographic printing	8	Bladder	no	NA	NA
71-43-2	Benzene	Production of plastics, resins, nylon fibers, dyes, detergents, gasoline	5, 8, 9	Acute myeloid leukaemia/acute non-lymphocytic leukaemia	no	1400	72
71-43-2	Benzidine (and benzidine-based dyes)	Azo dyes for wool, cotton, leather	3, 8	Bladder	yes	7	NA
50-32-8	Benzo[a]pyrene (Polycyclic aromatic hydrocarbon - PAH)	Coal industry, incomplete combustion of organic material	3, 8	Lung and multiple sites	no	980	NA
7440-41-7	Beryllium and beryllium compounds	Used in the production of alloys for aerospace, automotive, or biomedical applications	2, 3, 8	Lung	no	67	108
542-88-1	Bis(chloromethyl)ether; chloromethyl methyl ether	Chemical intermediates and alkylating agents	3, 8	Lung	no	2	NA
106-99-0	1,3-Butadiene	Production of synthetic rubbers and polymers	3, 8	Haematolymphatic organs	no	32	18

Continues on the next page ... ...

CAS	Carcinogenic agent	Some uses (or remarks)	ISCO	Cancer site (or type)	Banned in the EU	Exposed EU 1990-3 (thousands)	Deaths UK
7440-43-9	Cadmium and cadmium compounds	Production of nickel-cadmium batteries, pigments, coatings and platin, stabilizers for plastics	3, 8, 9	Lung	no	210	144
18540-29-9	Chromium (VI) compounds	Pigment for textile dyes, paints, inks and plastics, corrosion inhibitors, wood preservatives, leather tanning	3, 6, 8	Lung	no	800	1170
	Coal gasification (exposure to PAH, asbestos, silica, amines, arsenic, cadmium)	Production of fuel gas	3, 8	Lung	no	NA	NA
8007-45-2	Coal-tar distillation	Coal tar: anti-corrosion coatings, wood preservatives, binders for electrodes	3, 8	Skin, scrotum	no	NA	NA
65996-93-2	Coal-tar pitch	Electrode manufacturing, roofing, paving	3, 8	Lung	no	NA	72
	Coke production	As fuel in steel production	3, 8	Lung	no	NA	NA
	Engine exhaust, diesel	Exposure to benzene, PAHs, and other gaseous and particulate substances	2, 3, 5, 8, 9	Lung	no	3000	11736
75-21-8	Ethylene oxide	Raw material for production of mono-ethylene glycol (agrochemicals, plastics, pharmaceuticals, textiles)	2, 3, 6, 7, 8	Lymphoid tumours	no	47	0
50-00-0	Formaldehyde	Production of various types of resin used in the wood, plastic and other industries	2, 8, 9	Nasopharynx, leukaemia	no	990	144
	Ionizing radiation (X- and gamma-radiation including Radon-222 and its decay products)	Nuclear power production and fuel recycling, military activities, industrial operations, flying and medical procedures	2, 3, 5, 8	Salivary gland, oesophagus, stomach, colon, lung, bone, basal cell of the skin, female breast, kidney, bladder, brain and CNS, thyroid and leukaemia	no	2843	3366
	Iron and steel founding (occupational exposure during)Methoxsalen (8-methoxypsoralen) plus ultraviolet radiation	Exposure to various genotoxic and carcinogenic substances including PAHs, metals, formaldehyde	3, 8	Lung	NA	NA	450
	Leather dust	Shoe manufacture and repair. Exposure to various carcinogenic agents	7,8	Nasal cavity, paranasal sinuses	no	NA	180
	Magenta production	Manufacture of dyes	8	Bladder	no	NA	NA

Continues on the next page ... . . .

CAS	Carcinogenic agent	Some uses (or remarks)	ISCO	Cancer site (or type)	Banned in the EU	Exposed EU 1990-3 (thousands)	Deaths UK
101-14-4	4,4'-Methylenebis(2-chloroaniline) (MOCA)	Curing agent for polyurethane pre-polymers	8	Bladder	no	NA	NA
	Mineral oils, untreated or mildly treated	Lubricants, agricultural spray oils, printing inks	8	Skin (scrotum)	no	NA	10188
91-569-8	2-Naphthylamine	Rubber antioxidant, dye intermediate	8	Bladder	yes	2	NA
	Nickel compounds	Used to form alloys, electroplatin ceramics, pigments, stainless steel, nickel-cadmium batteries	3, 8	Lung, nasal cavity, paranasal sinuses	no	560	162
	Painter (occupational exposure as a)	Exposure to various genotoxic and carcinogenic substances including PAHs, azo dyes, benzene	7	Mesothelioma, bladder, lung	NA	NA	6012
1336-36-3	Polychlorinated biphenyls	Exposure in buildings and old devices contaminated with PCB	8, 9	Melanoma	yes	NA	0
	Rubber manufacturing industry	Exposure to various carcinogenic substances including PAHs, 2-naphthylamine	3, 8	Leukaemia, lymphoma, bladder, lung, stomach	NA	NA	90
68308-34-9	Shale oils	As fuel and electricity generation	8	Skin (scrotum)	no	NA	NA
14808-60-7	Silica dust, crystalline, in the form of quartz or cristobalite	Manufacture of glass, ceramics, hydraulic fracturing applications	3, 7, 8	Lung	no	3200	14202
	Solar radiation	Exposure to ultraviolet radiation (100-400 nm wavelength) from solar and artificial sources such as tanning devices	5, 9	Cutaneous malignant melanoma, squamus cell carcinoma of the skin, basal cell carcinoma of the skin	NA	9100	216
	Soot (as found in occupational exposure of chimney sweeps)	By-product of combustion of organic materials	7	Skin (scrotum), lung	no	NA	NA
1746-01-6	2,3,7,8-Tetrachlorodibenzo-para-dioxin	It has no known commercial applications. It may be produced during incineration, in metal processing, and in the bleching of paper pulp with free chlorine.	3, 8, 9	All cancers combined		NA	4158
	Tobacco smoke, second-hand	Exposure to carcinogenic substances such as benzo[a]pyrene, 4-aminobiphenyl, benzene, ethylene oxide, N'-Nitrosonornicotine		Lung	yes	7500	1062
95-53-4	ortho-Toluidine	Intermediate in the synthesis of herbicides and manufacture of dyes	3, 8	Bladder	no	NA	4482

Continues on the next page ... . . .

CAS	Carcinogenic agent	Some uses (or remarks)	ISCO	Cancer site (or type)	Banned in the EU	Exposed EU 1990-3 (thousands)	Deaths UK
79-01-6	Trichloroethylene	Used in metal cleaning or degreasing, and as a solvent in the rubbers industry	3, 8	Kidney	no	NA	72
75-01-4	Vinyl chloride	Manufacture of polyvinyl chloride (PVC), floor coverings	3, 8	Liver	no	40	54
	Wood dust	By-product of wood processing	7, 8, 9	Nasal cavity, paranasal sinuses, nasopharynx	no	2600	342

B . List of sensitising substances

Table 2: Sensitising substances. Sources: [9, 4, 5, 6, 10]

CAS	Sensitiser	Type	Somes uses (or remarks)	Isco	TTB
	Acrylic esters and methacrylates, various compounds	Sh	Acrylic esters are used commonly for the production of polymers. TTB for the precursor acrylic acid	3, 8	1,000,000 - 10,000,000
101-54-2	N 4-Aminodiphenylamine	Sh	Used as a dye intermediate and in the production of rubber	3, 8	10,000 - 100,000
141-43-5	2-Aminoethanol	Sh	Intermediate for the production of aziridines used in coatings, water treatment, and in the petroleum industries	2, 3, 8	100,000 - 1,000,000
2855-13-2	3-Aminomethyl-3,5,5-trimethyl-cyclohexylamine (Isophorone diamine)	Sh	Used as chemical intermediate in the production of hardeners for epoxy resins and coatings	3, 8	10,000 - 100,000
123-30-8	p-Aminophenol	Sh	Component of oxidative hair dyes	3, 5, 8	1,000 - 10,000
9000-90-2	a-Amylase	Sa	Enzyme used in fermentation processes and pharmaceuticals	3, 6, 8	1,000 - 10,000
122-40-7	a-Amylcinnamaldehyde	Sh	Used in perfumery and flavouring	3, 5, 8	NA
62-53-3	Aniline (including aminoazobenzenes)	Sh	One of the most important building blocks in chemistry. It is used in the manufacture of rubber, dyes and pigments, agricultural chemicals and pharmaceuticals	3, 6, 8, 9	1,000,000 - 10,000,000

Continues on the next page ... ...

CAS	Sensitiser	Type	Somes uses (or remarks)	Isco	TTB
	Animal hair, epithelia and other materials derived from animals	Sah		3, 6, 7, 8	NA
	Antibiotics, various	Sah		2, 3, 8	NA
17804-35-2	Benomyl	Sh	Fungicide	3, 6, 8	NA
2634-33-5	1,2-Benzisothiazol-3(2H)-one	Sh	Microbicide and fungicide	3, 8	NA
14548-60-8	Benzyl alcohol mono(poly)hemiformal	Sh	Benzyl alcohol is used as a chemical intermediate, in parfumery and flavoring, in textiles and plastics, cosmetics and pharmaceuticals	2, 3, 8	NA
	Beryllium and its compounds	Sah	Nuclear absorbents, aircraft engines, guidance systems and brakes; X-ray tube windows, turbine reactor blades, dental castings and prostheses, tool and die, sporting goods, home appliances, integrated circuits	2, 3, 8	NA
25068-38-6	Bisphenol-A-Epichlorhydrin Epoxy resin	Sh	Epoxy resins are used widely in industrial applications such as metal can coating, automotive primer, printed circuit boards, semiconductor encapsulants, adhesives, and aerospace composites	2, 3, 8	100,000 - 1,000,000
52-51-7	2-Bromo-2-nitro-1,3-propanediol	Sh	Used as an antiseptic and an antimicrobial agent for cosmetic preservation	3, 8	100 - 1,000
96-29-7	Butanone oxime	Sh	Used as an anti-skinning agent in paints and lacquers, and as a blocking agent for isocyanates in polyurethanes	3, 8	1,000 - 10,000
2426-08-6	n-Butyl glycidyl ether	Sh	Epoxy used as viscosity reducer	3, 8	NA
110-65-6	Butynediol	Sh	Added to electroplating baths as brightening agent	3, 8	100000
9012-54-8	Cellulases	Sa	Used in the pharmaceutical, food and paper industry	3, 8	1,000 - 10,000
	Cereal flour dusts (wheat, rye)	Sa		6, 7, 8	NA
79-07-2	2-Chloroacetamide	Sh	Used as a biocide, as a preservative and antiseptic in cosmetics, and as an intermediate in the production of herbicides and insecticides	3, 6, 8	100 - 1,000
96-34-4	Chloroacetic acid methyl ester	Sh	Solvent and intermediate	3, 8	1,000 - 10,000
108-42-9	m-Chloroaniline	Sh	Used as an intermediate for pesticides, pharmaceuticals, and dyes	3, 6, 8	1-10
59-50-7	p-Chloro-m-cresol	Sh	External germicides, preservative for glues, gums, paints, inks, textile and leather goods	3, 7, 8	1-10

Continues on the next page ... . . .

CAS	Sensitiser	Type	Somes uses (or remarks)	Isco	TTB
1897-45-6	Chlorothalonil	Sh	Used as a fungicide on vegetables, trees, fruits, turf, ornamentals, and other crops, as a wood preservative, as a biocide in paint	3, 6, 7, 8	NA
50-53-3	Chlorpromazine (2-Chloro-10-(3-dimethylaminopropyl)phenothiazine)	Sp	Antiemetic and antipsychotic drug in humans, and as tranquilizers in animals	2, 3, 8	NA
	Chromium(III) and (IV) compounds, soluble	Sh	Chromium and its compounds are used in metal alloys such as stainless steel, protective coatings on metal; magnetic tapes, and pigments for paints, cement, paper, rubber, composition floor covering and other materials	3, 7, 8, 9	NA
104-55-2	Cinnamaldehyde	Sh	Used as a brightener ion electroplating processes, as an animal repellent, as a flavourant in the food and perfume industry, in medical and the rubber industry	2, 3, 5, 8	100 - 1,000
104-54-1	Cinnamyl alcohol	Sh	Used in the cosmetic industry in soaps, lotions, fragrances	3, 8	1 - 10
513-79-1	Cobalt carbonat	Sah	Used in ceramics, manufacture of cobalt pigments and cobalt compounds, trace element added to solis, catalyst	3, 6, 8	1,000 - 10,000
420-04-2	Cyanamide	Sh	Used in agrochemical, pharmaceutical and industrial applications	3, 6, 8	1,000 - 10,000
95-33-0	N-Cyclohexyl-2-benzothiazolesulfenamide	Sh	Accelerator for rubber vulcanization	3, 8	10000
101-77-9	4,4'-Diaminodiphenylmethane	Sh	Used as an intermediate in the production of isocyanates, for preparation of plyurethane foams, spandex fibres, as a curing agent for epoxy resins, in the preparation of azo dyes	3, 8	10,000 - 100,000
120-78-5	N 2,2'-Dibenzothiazyl disulfide	Sh	Accelerator for natural rubber and synthetic isoprene rubber	3, 8	10,000 - 100,000
10222-01-2	2,2-Dibromo-2-cyanacetamide	Sh	Algecide, bactericide and fungicide. Used in the paper industry and industrial cooling systems	3, 8	NA
35691-65-7	1,2-Dibromo-2,4-dicyanobutane	Sh	Preservative for latex paints, adhesives, dispersed pigments, joint cements, and as a microbiocide	3, 5, 8	NA
542-75-6	1,3-Dichloropropene (cis and trans)	Sh	Pesticide	3, 6, 8	NA
111-42-2	Diethanolamine	Sh	Production of lubricants for the textile industry, as intermediate in the rubber industry, as a humectant and softening agent, and in the agricultural, cosmetics and pharmaceutical industry	3, 6, 8	100,000 - 1,000,000
111-40-0	Diethylenetriamine	Sh	Used as solvent, in the textile and military industry, as a component of epoxy resins for coatings	0, 3, 8	10000

Continues on the next page ... ...

CAS	Sensitiser	Type	Somes uses (or remarks)	Isco	TTB
793-24-8	N N-(1,3-Dimethylbutyl)-N'-phenyl-p-phenylenediamine	Sh	Antioxidant and antiozonant in the rubber industry	3, 8	10,000 - 100,000
57-14-7	1,1-Dimethylhydrazine	Sh	In the production of rocket fuel, in photography, and plant growth control agent	0, 3, 6, 8	1 - 10
6440-58-0	1,3-Dimethylol-5,5-dimethyl hydantoin	Sh	Used as a preservative in cosmetic products, in the control of bacteria and fungi in liquid detergents and household cleaning products	3, 8	100 - 1,000
94-37-1	Dipentamethylenethiuram disulfide	Sh	Vulcanization accelerator in the rubber industry	3, 8	NA
74-31-7	N N,N-Diphenyl-p-phenylenediamine	Sh	Antioxidant for rubber, polyethylene, feedstuffs, and vegetable oils	3, 6, 8	NA
68516-81-4	Disperse blue 106/124	Sh	Dyeing substance	3, 8	NA
730-40-5	Disperse Orange 3	Sh	Dyeing substance	3, 8	NA
2872-52-8	Disperse Red 1	Sh	Dyeing substance	3, 8	NA
3179-89-3	Disperse Red 17	Sh	Dyeing substance	3, 8	NA
2832-40-8	Disperse Yellow 3	Sh	Dyeing substance in textiles, sheepskins, furs, and for colouring nylon	3, 8	NA
97-77-8	Disulfiram	Sh	Vulcanization accelerator in the rubber industry	3, 8	1,000 - 10,000
7747-35-5	5-Ethyl-3,7-dioxa-1-azabicyclooctane	Sh	Used in leather tanning, as a dye, finishing, impregnation and care products	3, 7, 8	100 - 1,000
107-15-3	Ethylenediamine	Sah	Solvent for casein, albumin, and sulfur, emulsifier, stabilising rubber latex. Used in the preparation of dyes, synthetic waxes, resins, asphalt wetting agents, and as a herbicide	3, 8, 9	10000
97-53-0	Eugenol	Sh	Insecticide; used in perfumes, essential oils, analgesics, as flavorant	3, 5, 6, 8	100 - 1,000
4602-84-0	Farnesol	Sh	Used in perfumery and flavouring	3, 5, 8	NA
50-00-0	Formaldehyde	Sh	Used as germicide, in the production of resins, wood products, plastics, fertilizers and foam insulation, in the textile industry, as a preservative and disinfectant, and as a hardening agent	2, 3, 5, 6, 8	1000000
	Fragrance components	Sh	Widely differing substances (e.g. Balsam of Peru). Difficulties of assessing occupational exposure	2, 4, 5, 8	NA
106-24-1	Geraniol	Sh	Frequently used fragrance material used in flowery-roselike compositions and favour compositions in the food industry, in soaps and cosmetics	3, 5, 8	1,000 - 10,000
111-30-8	Glutaraldehyde	Sah	Used in histology procedures, in fish farming to control viruses and micro-organisms, in cosmetics as a preservative, and in the textile industry	2, 3, 6, 8	1000

Continues on the next page ... . . .

CAS	Sensitiser	Type	Somes uses (or remarks)	Isco	TTB
30618-84-9	Glyceryl monothioglycolate	Sh	Used in permanent wave solutions in hairdressing	3, 5, 8	10 - 100
107-22-2	Glyoxal	Sh	Used in the paper industry for sizing, washable wall paper, treating glue surface of envelopes, in textile industry for preventing shrinking and creasing, and as a substitute for formaldehyde in embalming fluids	3, 5, 8	10,000 - 100,000
	Gold and its inorganic compounds	Sh	Gold compounds are used as electrolyte in the electroplating industry, in the manufacture of ruby glass, coloured enamels, and painting porcelain; in photography, gold plating, special inks, medicine	2, 3, 7, 8	NA
12070-12-1	Hard metal containing tungsten carbide and cobalt (inhalable fraction)	Sah	Tungsten carbide is used in cemented carbide tools, dies, wear-resistant parts, cements, electrical resistors, and abrasive in liquids	3, 8, 9	10,000 - 100,000
85-42-7	Hexahydrophthalic anhydride	Sa	Intermediate for alkyds, plasticizers, insect repellents, and rust inhibitors, and hardener in epoxy resins	3, 8	10,000 - 100,000
822-06-0	1,6-Hexamethylene diisocyanate	Sah	Production of polyurethanes, which are processed to adhesives, insulating foams, lacquers, and foams	3, 7, 8, 9	10,000 - 100,000
100-97-0	Hexamethylenetetramine	Sh	Used in adhesives, coatings, sealing compounds, in the chemical detection of metals, for vulcanizing rubber, as corrosion inhibitor for steel, as dye fixative, and antimicrobial food additive	3, 8	10,000 - 100,000
302-01-2	Hydrazine	Sh	Most important uses are as polymerization initiators	3, 8	10,000 - 100,000
123-31-9	Hydroquinone	Sh	Used in photographic applications, rubber industry, monomer inhibitors, dyes, pigments, agricultural chemicals	3, 6, 8	10,000 - 100,000
107-75-5	Hydroxycitronellal	Sh	Blender used in perfumery	3, 5, 8	NA
7803-49-8	Hydroxylamine and its salts	Sh	Reducing agent in photography, synthetic and analytical chemistry. Used to purify ketones, as antioxidant for fatty acids and soaps, as deharing agent for hides	3, 5, 8, 9	1,000 - 10,000
31906-04-4	4-(4-Hydroxy-4-methyl pentyl)-3-cyclohexene-1-carboxaldehyde	Sh	Used in fragrance compositions	3, 5, 8	NA
55406-53-6	3-Iodo-2-propynyl butylcarbamate	Sh	Fungicide and antimicrobial used in industry and residential settings. Used in the manufacturing of paints, adhesives, in metal cutting and drilling fluids,in paper coating, canvas and wood products manufacturing	3, 5, 8	NA

Continues on the next page ... . . .

CAS	Sensitiser	Type	Somes uses (or remarks)	Isco	TTB
97-54-1	Isoeugenol	Sh	Manufacture of vanillin for the food industry. Used also as a fragrance in household laundry, cleaning products especially laundry powders and liquids, dish-washing liquids, hard surface cleaning products, and toilet cleansing products	3, 5, 8, 9	NA
4098-71-9	Isophorone diisocyanate	Sah	Production of polyurethanes, raw material for polyurethane paints, in industrial coating applications, and electrostatic powder coatings	3, 8	10,000 - 100,000
101-72-4	N-Isopropyl-N'-phenyl-p-phenylenediamine	Sh	Protection of rubbers against oxidation, ozone, flex-cracking	3, 8	1,000 - 10,000
138-86-3	D,L-Limonene and similar mixtures	Sh	Various applications in the food and cosmetics industry and as a solvent	3, 8	NA
108-31-6	Maleic anhydride	Sah	Manufacture of resins, dye intermediates, in pharmaceuticals, agricultural chemicals	3, 6, 8	1,000 - 10,000
12427-38-2	Manganous ethylenebis(dithiocarbamate)	Sh	Fungicide for vegetables, for seed treatment of vegetables and field crops, for deciduous fruits and nuts	3, 6, 8	NA
129-16-8	Merbromin	Sh	Antibacterial and antiseptic	2, 3, 8	NA
149-30-4	2-Mercaptobenzothiazole	Sh	Vulcanization accelerator for rubber and corrosion inhibitor	3, 8	1,000 - 10,000
	Mercury (metallic mercury and inorganic mercury compounds)	Sh	Mercury compounds are used in treating seeds for fungi and seedborne diseases, as timber preservatives, and disinfectants in batteries (mercuric oxide), pigments, catalysts, explosives, in laboratory-based research, and in some pharmaceutical applications	2, 3, 6, 8	NA
4080-31-3	Methenamin 3-chlorallylchloride	Sh	Used in pesticides, industrial adhesives and coatings, textiles, cosmetics, soaps, hair care products	3, 5, 8	NA
66204-44-2	N,N'-Methylene-bis(5-methyloxazolidine)	Sh	Metal working fluid	3, 8	NA
101-68-8	4,4'-Methylene diphenyl diisocyanate	Sah	Used primarily to make rigid and semi-rigid polyurethane foams	3, 8	100,000 - 1,000,000
2682-20-4	N 2-Methyl-4-isothiazolin-3-one	Sh	Used as a biocide and preservative	2, 3, 8, 9	NA
11070-44-3	Methyltetrahydrophthalic anhydride	Sa	Hardener for Epoxy Resins	3, 8	10,000 - 100,000
479-45-8	N-Methyl-2,4,6-N-tetranitroaniline	Sh	Used as an explosive in detonators and primers	0, 3, 8	NA
78-94-4	Methyl vinyl ketone	Sh	Used as a starting material for plastics; as intermediate in synthesis of steroids and vitamin A	3, 8	NA

Continues on the next page ... . . .

CAS	Sensitiser	Type	Somes uses (or remarks)	Isco	TTB
	N Microbial rennets: endothiapepsin and mucorpepsin	Sa	Rennets are enzymes used to make cheese and custards and as a therapeutic digestive enzyme	3, 6, 8	NA
60-34-4	Monomethylhydrazine	Sh	Used as an antibiotic, as an chemical intermediate, and in rocket fuel	0, 3, 8	100 - 1,000
102-77-2	N 2-(4-Morpholinylmercapto)benzothiazole	Sh	Used as a delayed action vulcanization accelerator	3, 8	1,000 - 10,000
300-76-5	Naled	Sh	Insecticide used for numerous crops and in food processing plants	3, 6, 8	NA
3173-72-6	1,5-Naphthylene diisocyanate	Sa	Used in painting, and in the production of plastics	3, 8	1,000 - 10,000
9006-04-6	Natural rubber latex	Sah	Frequent exposure when using latex-based gloves	2, 5, 6, 9	NA
	Nickel and nickel compounds	Sah	Used to form alloys, electroplatin ceramics, pigments, stainless steel, nickel-cadmium batteries. TBB for nickel	3, 8	NA
91-29-2	4-Nitro-4'-aminodiphenylamine-2-sulfonic acid	Sh	Intermediate in the manufacture of dyes	3, 8	100 - 1,000
5307-14-2	2-Nitro-p-phenylenediamine	Sh	In dyeing furs brown and reddish brown, and as dye intermediate in semi-permanent and permanent hair dye formulations	3, 5, 8	NA
	Oakmoss extracts	Sh	Used in the production of perfumes	3, 5, 8	NA
26530-20-1	2-Octyl-4-isothiazolin-3-one	Sh	Fugicide used in wood coatings	3, 5, 7, 8	NA
23696-28-8	Olaquindox (N-(2-Hydroxyethyl)-3-methyl-2-quinoxalinecarboxamide)	SP	Animal feed additive	3, 6, 8	NA
7647-10-1	Palladium chloride and other bioavailable palladium(II) compounds	Sh	Use in toning solutions, photography, electroplating parts of clocks and watches; manufacture of indelible ink	3, 8	NA
9001-73-4	Papain	Sa	Used in the food and textile industry	3, 8	NA
9001-75-6	Pepsin	Sa	Used in the food and textile industry and chemistry	2, 3, 8	NA
108-45-2	m-Phenylenediamine	Sh	Used as the starting material for many dyes	3, 8	1,000 - 10,000
106-50-3	p-Phenylenediamine	Sh	Used for reinforcing tires and V-belts, as substitute for asbestos in brake linings, and for aerospace and sporting applications	2, 3, 8	10,000 - 100,000
90-30-2	N-Phenyl-1-naphthylamine	Sh	Used in the synthesis of/ dyes and other organic chemicals, and as rubber antioxidant	3, 8	100 - 1,000
135-88-6	N-Phenyl-2-naphthylamine	Sh	Rubber antioxidant, lubricant and inhibitor	3, 8	NA

Continues on the next page ... . . .

CAS	Sensitiser	Type	Somes uses (or remarks)	Isco	TTB
85-44-9	Phthalic anhydride	Sa	Use in the production of alkyd resins, plasticizers, as hardener for resins, polyesters, for synthesis of dyes, chlorinated products, pharmaceutical intermediates, insecticides,and as laboratory reagent	2, 3, 6, 8	100,000 - 1,000,000
	Phytases	Sa	Used in the food industry	3, 6, 8	NA
88-89-1	Picric acid	Sh	Uses in the production of explosives, matches and electric batteries, in the leather industry, and for the manufacture of colored glass	0, 3, 8, 9	NA
88-88-0	Picryl chloride	Sh	Used for the production of acrylate adhesives, epoxy resin, concrete and gypsum impregnation	3, 8	1,000 - 10,000
110-85-0	Piperazine	Sah	Used as a scruber in gas-washer formulations, as a hardener for glue prepolymersand, as a raw material to prepare intermediates used for antihistamine production, and in the manufacture of insecticides and fibers	3, 8	1000
	Platinum compounds	Sah	Used in automobile catalytic converters	3, 7, 8	NA
9016-87-9	?Polymeric MDI?	Sah	Used as a binder resind for particle board	3, 8	NA
75-56-9	N Propylene oxide	Sh	Used in the production of polyurethanes, lubricants, oil demusifiers, as a solvent, herbicide, soil sterilant	3, 6, 8	1000000
8003-34-7	Pyrethrum	Sh	Insecticide	3, 6, 8	NA
106-51-4	Quinone	Sh	Used in the tanning of hides, making of gelatine insol, and strenghtening of animal fibers	3, 8	Intermediate Use Only
108-46-3	Resorcinol	Sh	Used in In tanning; manufacturing resins, resin adhesives, hexylresorcinol, p-amino salicyclic acid, explosives, and dyes	0, 3, 6, 8, 9	10,000 - 100,000
8050-09-7	Rosin	Sh	Natural resin with widely varying industrial applications such as in printing, paper industry, food and clothing industry	3, 8	100,000 - 1,000,000
9006-04-6	Rubber components (e.g. Dithiocarbamates, p-Phenylenediamine)	Sh	Natural rubber is used in the production of pneumatic tires, rubber footware, inflatable and sectional seals; elastic thread and tapefloor coveringsadhesives and solution	2, 3, 7, 8	NA
148-18-5	Sodium diethyldithiocarbamate	Sh	Corrosive inhibitor, rubber accelerator, intermediate	3, 8	1,000 - 10,000
	Soya bean constituents	Sa		3, 6, 8	NA
9014-01-1	Subtilisins	Sa	Laundry detergent and automatic dishwashing for removal of proteinaceous stains. Animal feed for improved digestibility of proteins in animal feed. New industrial use for membrane cleaning	3, 6, 8, 9	1,000 - 10,000

Continues on the next page ... ...

CAS	Sensitiser	Type	Somes uses (or remarks)	Isco	TTB
8002-26-4	Tall oil, distilled	Sh	Used in the rubber and adhesive industry, in linoleum, soaps, fungicides, asphalt emulsions, and cutting oils	3, 8	10,000 - 100,000
54-64-8	Thimerosal	Sh	Used as an ophthalmic preservative, a topical anti-infective, as a fungicide for cotton seed treatment, and as antimicrobial preservative in cosmetics	3, 6, 8	NA
68-11-1	N Thioglycolic acid	Sh	Reagent for iron, manufacture of thioglycolates, permanent-wave solutions and depilatories, vinyl stabilizer, manufacture of pharmaceuticals	2, 3, 5, 7	10,000 - 100,000
62-56-6	Thiourea	Sh SP	Photographic toning agent, flame-retardant textile sizes and flame-retardant finish for cotton. Used for flame resistance and improved handling properties for nylon-based products. Nitrification inhibitor for ammonia-based fertilizer; intermediate for pesticides and fungicides	3, 6, 8	100 - 1,000
137-26-8	Thiram	Sh	Used as a fungicide in agriculture and food storage	6	1,000 - 10,000
95-80-7	Toluene-2,4-diamine	Sh	Used in the production diisocyanates for the polyurethane industry	3, 8	Intermediate Use Only
106-49-0	p-Toluidine	Sh	Used as an intermediate in the production of different azo-pigments, quinacridone-pigments and paper dyes, pesticides and pharmaceuticals	3, 8	10,000 - 100,000
112-24-3	Triethylenetetramine	Sh	Used as a chemical intermediate for polyamide resins, imidazoline, and aminoamide surfactants, and as a hardener for epoxy resins	3, 8	NA
126-71-6	Triisobutyl phosphate	Sh	Solvent used in the manufacture of paper and textiles	3, 8	1,000 - 10,000
552-30-7	Trimellitic anhydride	Sa	Plasticizer for polyvinyl chloride, high-temperature plastics, wire insulation, gaskets, automotive upholstery, dyes, resins, and printing inks	3, 8	10,000 - 100,000
118-96-7	2,4,6-Trinitrotoluene	Sh	Used as a high explosive in military and industrial applications	0, 2, 3, 8, 9	10,000 - 100,000
603-35-0	Triphenyl phosphine	Sh	Used as an intermediate in pharmaceuticals, in organic synthesis and polymerization initiator	3, 8	100
4719-04-4	2,2',2"-(hexahydro-1,3,5-triazine-1,3,5-triyl)triethanol	Sh	Used as a formaldehyde-releasing biocide in metalworking fluids, as an antimicrobial used to preserve adhesives, metalworking fluids, indoor construction materials, lubricants, aqueous mineral slurries, paints, stains, coatings, fuel and oil in storage, oil field drilling muds, inks and dyes, chemical and clinical reagents, industrial water systems, and household and industrial cleansers and detergents	3, 7, 8, 9	10,000 - 100,000

Continues on the next page ... . . .

CAS	Sensitiser	Type	Somes uses (or remarks)	Isco	TTB
8006-64-2	Turpentine	Sh	Solvent or dilution agent for oils, coatings, resins, paints and floor and shoe polishes.	2, 3, 8, 9	10,000 - 100,000
1484-13-5	Vinylcarbazole	Sh	Used in the plastic industry	3, 8	NA
	Woods, various species	Sh	The processing of woods includes a wide variety of industries such as sawmills, plywood mills, wood composites production, furniture factories and small workshops	3, 7, 8, 9	NA
37278-89-0	Xylanases	Sa	Used in the production of animal feed, in the cellulose and paper industry, and in the starch and bread-making industries	3, 6, 7, 8	NA
1477-55-0	m-Xylylenediamine	Sh	Curing agent for epoxy resins	3, 8	1,000 - 10,000
13530-65-9	Zinc chromate	Sh	Used in priming paints for metals, artists' colours, pigment in automotive parts	2, 3, 8	NA
	Zirconium and its insoluble and soluble compounds	Sah	Used in cast iron and steel manufacturing, as pacifier and polishing powder for lens and television tubes, in arc lamps; pigment in plastics, making rayon spinnerets; in superconductive magnets; in making surgical appliances, and in nuclear reactors	2, 3, 8	NA

C . List of mutagenic and reprotoxic substances

Table 3: Mutagenic and reprotoxic substances. Sources: [9, 11, 4, 5, 6, 12, 10].

CAS	Agent	Somes uses (or remarks)	M	RF	RE	ISCO	TTB
67-64-1	Acetone	Solvent for fats, oils, waxes, resins, rubber, plastics, lacquers, varnishes, rubber cements	NA	NA	B	3, 7, 8	10,000 - 100,000
35554-44-0	1-(2-Allyloxy)-2-(2,4-dichlorphenyl)ethyl)-1H-imidazol	Fungicide in agriculture, as a disinfectant for stable and kennel equipment	NA	NA	B	3, 6, 8, 9	NA
1464-53-5	2,2'-Bioxirane	Used for curing polymers, cross-linking textile fibers, and producing erythritol and pharmaceuticals	2	3	NA	3, 8	NA
112-49-2	1,2-Bis(2-methoxyethoxy)ethane	Used as a solvent for gases and coupling immiscible liquid	NA	2	2	3, 8	10 - 100

Continues on the next page ... . . .

CAS	Agent	Somes uses (or remarks)	M	RF	RE	ISCO	TTB
56-35-9	Bis(tri-n-butyltin)oxide	Used as an antimicrobial, disinfectant for hard surfaces, as sanitizer for laundry, in water-based paints and in adhesives, as preservative for wood, paper, textiles, and as pesticide	NA	2	3	3, 6, 7, 8	Intermediate Use Only
10043-35-3	Boric acid	Hydraulic fracturing fluid; insecticide; used for weatherproofing wood and fireproofing fabrics; as a preservative; manufacture of cements, crockery, porcelain, enamels, glass, borates, leather, carpets, hats, soaps; printing and dyeing, paiting	NA	NA	B	3, 6, 8, 9	100,000 - 1,000,000
151-67-7	2-Bromo-2-chloro-1,1,1-trifluoroethane	Used as a medication and anaesthetic during surgical procedures	NA	NA	2	2, 3, 8	NA
2426-08-06	1-n-Butoxy-2,3-epoxypropan	Reactive diluent for epoxy resins	2	NA	NA	3, 8	NA
688-73-3	Tri-n-butyltin hydride	Reducing agent for the conversion of alkyl halides to hydrocarbons	NA	NA	B	3, 8	NA
10605-21-7	Carbendazim	An agricultural chemical and fungicide	NA	NA	B	3, 6, 8, 9	NA
75-15-0	Carbon disulfide	Used in optical glass, paints, enamels, varnishes, paint removers, tallow, explosives, rocket fuel, putty preservatives, rubber cement; as a solvent for waxes, lacquers, camphor, resins, and vulcanized rubber	NA	NA	B	3, 7, 8, 9	100,000 - 1,000,000
630-08-0	Carbon monoxide	It is a product of the incomplete combustion of carbon-containing fuels and is also produced by natural processes. Reducing agent in metallurgical operations. Used in the production of several chemical intermediates	NA	NA	B	3, 8, 9	100,000 - 1,000,000
4080-31-3	N-(3-Chloroallyl)hexaminium chloride	Microbicide, industrial adhesives and coatings; resin, latex, and polymer emulsions; metalworking cutting fluids; oil recovery drilling muds/packer fluids; textiles fibers and cordage; preservative in cosmetics, hair care products, soaps, adhesives, joint cements	NA	NA	B	3, 6, 8, 9	NA
6147-53-1	Cobalt(II) acetate tetrahydrate	Used in sympathetic inks, feed additives, catalysts, foam stabilizers, driers for coatings, and anodizing	3	2	NA	3, 8	NA
513-79-1	Cobaltcarbonat	Used in ceramics, manufacture of Cobalt pigments, and preparation of Cobalt compounds	3	2	NA	3, 8	1,000 - 10,000
7646-79-9	Cobalt dichloride	Used as Invisible ink, humidity and water indicator; in hygrometers; temperature indicator in grinding; in electroplating; for painting on glass and porcelain, in the preparation of catalysts; as fertilizer and feed additive	3	2	NA	2, 3, 6, 8, 9	1,000 - 10,000

Continues on the next page ... . . .

CAS	Agent	Somes uses (or remarks)	M	RF	RE	ISCO	TTB
10026-22-9	Cobalt(II) nitrate hexahydrate	Used to make cobalt pigments, invisible inks, catalysts, and vitamin B12 supplements, and for decorating stoneware and porcelain	3	2	NA	3, 7, 8	NA
84-74-2	Dibutyl phthalat (DBP)	Used as a solvent for chlorinated rubber, plasticizer in nitrocellulose lacquers, elastomers, explosives, nail polish and solid rocket propellants; as a solvent for perfume oils; in adhesives and as insect repellent for textiles	NA	2	2	0, 3, 5, 7, 8, 9	1,000 - 10,000
111-96-6	Diethylene glycol dimethylether	Solvent in chemical reactions involving metals and organometallic compounds; used in the production of plastics	NA	NA	B	3, 8	100 - 1,000
127-19-5	N,N-Dimethylacetamide	Solvent for plastics, resins, gums, and electrolytes; catalyst; paint remover; high-purity solvent for crystallization and purification	NA	3	2	3, 8	10,000 - 100,000
68-12-2	N,N-Dimethylformamide	Solvent for liquids, gases, for orlon and similar polyacrylic fibers, pharmaceutical processing, leather fabrics, reagent and catalyst in organic chemistry	NA	NA	B	3, 7, 8	10,000 - 100,000
646-06-0	1,3-Dioxolane	Low-boiling solvent and extractant for oils, fats, waxes, dyes, and cellulose derivatives; co-monomer for manufacture of polyacetals and other polymers, solvent for chemical reactions	NA	NA	B	3, 8	1,000 - 10,000
110-80-5	2-Ethoxyethanol	Solvent for nitrocellulose, lacquers and dopes; in varnish removers, cleansing solutions, dye baths; finishing leather with water pigments and dye solutions; increasing stability of emulsions; solvent for epoxy and other coatings, printing inks, adhesives	NA	NA	B	3, 7, 8, 9	1,000 - 10,000
111-15-9	2-Ethoxyethyl acetate	In automobile lacquers to retard evaporation and impart high gloss, for nitrocellulose, oils, and resins, in wood stains and varnish removers, and in products for the treatment of textiles and leathers	NA	NA	B	3, 8, 9	NA
7439-92-1	Lead, compounds	Used in mining and smelting, in lead-acid bateries, crystal glass, in paints and ink, ammunition, cable and wire production, in bridges, ships, steel towers, in metal recycling	NA	3	1	3, 7, 8, 9	1,000,000 - 10,000,000
72-43-5	Methoxychlor	Insecticide used in agriculture, dairies, and in household and industrial premises	NA	NA	B	3, 6, 8	NA
625-45-6	Methoxyacetic acid	Used in organic synthesis in the chemical industry	NA	NA	B	3, 8	Intermediate Use Only
109-86-4	2-Methoxyethanol	Solvent for low viscosity cellulose acetate, natural resins, some synthetic resins; used in dyeing leather and in quick drying varnishes, enamels, nail polishes, wood stains	NA	NA	B	3, 7, 8	1,000 - 10,000

Continues on the next page ... ...

CAS	Agent	Somes uses (or remarks)	M	RF	RE	ISCO	TTB
110-49-6	2-Methodyethylacetat	Industial solvent for various gums, resins, waxes, oils; textile printing; photographic film; lacquers; dopes	NA	NA	B	3, 7, 8	Intermediate Use Only
1589-47-5	2-Methoxypropanol-1	It is a byproduct during the synthesis of 1-methoxy-2-hydroxypropane	NA	NA	B	3, 8	NA
70657-70-4	2-Methoxypropyl acetate	Permitted for use as an inert ingredient in non-food pesticide products; solvent	NA	NA	B	3, 8, 9	NA
74-87-3	Methyl chloride	Foaming agent in plastics industry, refrigerant, fluid for thermometric and thermostatic equipment, methylating agent in organic synthesis, herbicide	NA	NA	B	3, 6, 8, 9	1,000,000 - 10,000,000
23696-28-8	Olaquindox	Used as veterinary growth stimulant	2	3	NA	3, 6, 8	NA
335-67-1	Perfluorooctanoic acid	Used in fire-fighting applications, cosmetics, greases and lubricants, paints, polishes and adhesives	NA	NA	B	3, 5, 7, 8	NA
1763-23-1	Perfluorooctane sulfonic acid	Surfactant in fire fighting foam; surfactant for alkaline cleaners; emulsifier in floor polish; mist suppressant for metal plating baths; surfactant for etching acids for circuit boards; pesticide active ingredient for ant bait traps	NA	NA	B	3, 5, 7, 8	NA
62-74-8	Sodium fluoroacetate	Rodenticide, used also for control of wild rabbits, wild dogs and wild pigs	NA	NA	B	3, 8, 9	NA
3811-73-2	Sodium omadine	Used as biocide in metalworking, cutting, cooling, and lubricating fluids, latex emulsions, aqueous fiber lubricants and inks, laundry rinse additives and detergents, carpet cleaners, analytical and diagnostic reagents; also used as preservative for water based mixtures used in making concrete	NA	NA	B	3, 8, 9	Intermediate Use Only
	Tobacco smoke, second-hand	Exposure depends on tobacco-smoke regulation	3	NA	1	5, 9	NA
87-86-5	Pentachlorphenol	Insecticide for termite control; general herbicide. Antimicrobial preservative and fungicide for wood, textiles, paints, adhesives, leather, pulp, paper, industrial waste systems, and building materials	3	NA	2	3, 6, 7, 8, 9	NA
1336-36-3	Polychlorinated biphenyls	Exposure in buildings and old devices contaminated with PCB	NA	2	2	8, 9	NA
5216-25-1	alpha,alpha,alpha,4-Tetrachlorotoluene	Used as intermediate for pharmaceuticals, dyes, and other organic chemicals	NA	2	NA	3, 8	Intermediate Use Only
96-18-4	1,2,3-Trichlorpropane	Used as an intermediate in the production of polysulfone liquid polymers, dichloropropene and hexafluoropropylene	3	2	NA	3, 8	1,000 - 10,000

Continues on the next page ... ...

CAS	Agent	Somes uses (or remarks)	M	RF	RE	ISCO	TTB
112-27-6	Triethylene glycol	Bacteriostatic agent for air sanitization and deodorization. In compounds used as a fungicide, virucide, miticide and insecticide. As an inert ingredient facilitates delivery of formulated pesticides used as herbicides, fungicides, insecticides	NA	NA	B	3, 6, 8, 9	10,000 - 100,000
512-56-1	Trimethyl phosphate	Gasoline additive; methylating agent, intermediate in the production of polymethyl polyphosphates; flame retardant solvent for paints and polymers	2	NA	NA	3, 8, 9	NA
115-96-8	Tris(2-chlorethyl)phosphat	Used as an additive plasticiser and viscosity regulator with flame-retarding properties for polyurethane, polyesters, polyvinyl chloride and other polymers	NA	2	NA	3, 8	10 - 100
2451-62-9	1,3,5-Tris(oxiranylmethyl)-1,3,5-triazin-2,4,6(1H,3H,5H)-trione	Used in polyester powder coatings (paints)	2	3	NA	3, 8	100 - 1,000
81-81-2	Warfarin	Rodenticide applied in houses, animal and agricultural premises, and commercial and industrial sites	NA	NA	B	3, 6, 8, 9	Intermediate Use Only

D . List of pathogens

Table 4: Pathogens by species, risk group according to EU Directive 2000/54/EC, and occupational class. Reference: [13].

Pathogen	Classification	Type	ISCO
Aspergillus fumigatus	2	Fungus	3, 6, 8, 9
Bordetella pertussis	2	Bacterium	2, 3
Borrelia burgdorferi	2	Bacterium	5, 6, 9
Campylobacter jejuni	2	Bacterium	7
Campylobacter spp.	2	Bacterium	2, 6, 7, 9
Central European tick-borne encephalitis virus	3	Virus	6
Chlamydia trachomatis	2	Bacterium	5
Clostridium tetani	2	Bacterium	2, 6, 9
Coxiella burnetii	3	Bacterium	6

Continues on the next page ... . . .

Pathogen	Classification	Type	ISCO
Cryptosporidium parvum	2	Parasite	5
Cytomegalovirus	2	Virus	2, 5
Echinococcus multilocularis	3	Parasite	6
Escherichia coli, verocytotoxigenic strains	3	Bacterium	6, 7, 9
Escherichia coli (with the exception of non-pathogenic strains)	2	Bacterium	6, 7, 9
Francisella tularensis (Type A)	3	Bacterium	6
Francisella tularensis (Type B)	2	Bacterium	6
Giardia lamblia (Giardia intestinalis)	2	Parasite	3, 5
Helicobacter pylori	2	Bacterium	2, 3, 6, 9
Hepatitis A virus (human enterovirus type 72)	2	Virus	5, 9
Hepatitis B virus	3	Virus	2, 3
Hepatitis C virus	3	Virus	2, 3, 5, 9
Histoplasma capsulatum duboisii	3	Fungus	8
Human herpes virus 7	2	Virus	2, 3, 5
Human herpes virus 8	2	Virus	2, 3, 5
Human immunodeficiency viruses	3	Virus	2, 3, 5
Human papillomaviruses	2	Virus	5
Influenza viruses types A, B and C	2	Virus	2, 3, 5, 6, 7, 9
Legionella pneumophila	2	Bacterium	8
Leishmania spp.	2	Parasite	0
Leptospira interrogans (all serovars)	2	Bacterium	5, 6, 7, 9
Measles virus	2	Virus	2, 3
Mumps virus	2	Virus	2, 3
Mycobacterium bovis (except BCG strain)	3	Bacterium	2, 3, 6, 9
Mycobacterium chelonae	2	Bacterium	8
Mycobacterium tuberculosis	3	Bacterium	2, 3, 5, 9
Neisseria gonorrhoeae	2	Bacterium	5
Neisseria meningitidis	2	Bacterium	2, 3
Pasteurella multocida	2	Bacterium	2, 3
Pseudomonas aeruginosa	2	Bacterium	7
Rhinoviruses	2	Virus	3
Rickettsia conorii	3	Bacterium	6
Rubivirus	2	Virus	2, 3
Staphylococcus aureus	2	Bacterium	2, 3, 6, 7, 9
Streptococcus pyogenes	2	Bacterium	2, 3, 7

Continues on the next page ... . . .

Pathogen	Classification	Type	ISCO
Strongyloides stercoralis	2	Parasite	6, 9
Toxocara canis	2	Parasite	2, 5, 6, 7, 9
Toxoplasma gondii	2	Parasite	2, 5, 6, 9
Treponema pallidum	2	Bacterium	5
Varicella zoster virus	2	Virus	2
West Nile fever virus	3	Virus	3

References

[1] IARC (2012). *A Review of Human Carcinogens: Chemical Agents and Related Occupations. Volume 100F.* Lyon: IARC.

[2] IARC (2012). *A Review of Human Carcinogens: Radiation. Volume 100D.* Lyon: IARC.

[3] IARC (2012). *A Review of Human Carcinogens: Arsenic, Metals, Fibres, and Dusts. Volume 100C.* Lyon: IARC.

[4] Elvers, B. (Ed.) (2000). *Ullmann's Encyclopedia of Industrial Chemistry.* Wiley-VCH.

[5] National Library of Medicine, Hazardous Substances Data Bank. URL `http://toxnet.nlm.nih.gov/index.html`.

[6] National Library of Medicine, Haz-Map. URL `http://hazmap.nlm.nih.gov`.

[7] Kauppinen, T., Toikkanen, J., Pedersen, D., et al. (2000). Occupational exposure to carcinogens in the European Union. *Occupational and Environmental Medicine*, 57(1):10–18, doi:10.1136/oem.57.1.10.

[8] Rushton, L., Bagga, S., Bevan, R., et al. (2010), The burden of occupational cancer in Great Britain. URL `http://www.hse.gov.uk/cancer/`.

[9] Bundesanstalt für Arbeitsschutz und Arbeitsmedizin (2008), Technische Regeln für Gefahrstoffe TRGS 905. URL `http://www.baua.de/en`.

[10] European Chemicals Agency, Registered Substances under the REACH regulation. URL `http://echa.europa.eu/`.

[11] Bundesanstalt für Arbeitsschutz und Arbeitsmedizin (2004), Criteria for the Classification of Biological Agents TRBA 450. URL `http://www.baua.de/en`.

[12] Deutsche Forschungsgemeinschaft (DFG) (2013). *List of MAK and BAT Values 2013: Maximum Concentrations and Biological Tolerance Values at the Workplace.* Weinheim: Wiley, doi:10.1002/9783527675128.ch4.

[13] Haagsma, J.A., Tariq, L., Heederik, D.J., et al. (2012). Infectious disease risks associated with occupational exposure: a systematic review of the literature. *Occupational and environmental medicine*, 69(2):140–146, doi:10.1136/oemed-2011-100068.
